# Investigation of the Exometabolomic Profiles of Rat Islets of Langerhans Cultured in Microfluidic Biochip

**DOI:** 10.3390/metabo12121270

**Published:** 2022-12-15

**Authors:** Amal Essaouiba, Rachid Jellali, Françoise Gilard, Bertrand Gakière, Teru Okitsu, Cécile Legallais, Yasuyuki Sakai, Eric Leclerc

**Affiliations:** 1 Biomechanics and Bioengineering, CNRS, Université de Technologie de Compiègne, Centre de Recherche Royallieu CS 60319, 60203 Compiègne, France; 2 CNRS IRL 2820, Laboratory for Integrated Micro Mechatronic Systems, Institute of Industrial Science, University of Tokyo, 4-6-1 Komaba, Meguro-ku, Tokyo 153-8505, Japan; 3 Plateforme Métabolisme-Métabolome, Institute of Plant Sciences Paris-Saclay (IPS2), Université Paris-Saclay, CNRS, INRAE, Université Evry, Université Paris Cité, Bâtiment 360, Avenue des Sciences, 91190 Gif sur Yvette, France; 4 Institute of Industrial Science, University of Tokyo, 4-6-1 Komaba, Meguro-ku, Tokyo 153-8505, Japan; 5 Department of Chemical Engineering, Faculty of Engineering, University of Tokyo, 7-3-1 Hongo, Bunkyo-ku, Tokyo 113-8654, Japan

**Keywords:** microfluidic culture, biochip, islets of Langerhans, exometabolome, diabetes

## Abstract

Diabetes mellitus (DM) is a complex disease with high prevalence of comorbidity and mortality. DM is predicted to reach more than 700 million people by 2045. In recent years, several advanced in vitro models and analytical tools were developed to investigate the pancreatic tissue response to pathological situations and identify therapeutic solutions. Of all the in vitro promising models, cell culture in microfluidic biochip allows the reproduction of in-vivo-like micro-environments. Here, we cultured rat islets of Langerhans using dynamic cultures in microfluidic biochips. The dynamic cultures were compared to static islets cultures in Petri. The islets’ exometabolomic signatures, with and without GLP1 and isradipine treatments, were characterized by GC-MS. Compared to Petri, biochip culture contributes to maintaining high secretions of insulin, C-peptide and glucagon. The exometabolomic profiling revealed 22 and 18 metabolites differentially expressed between Petri and biochip on Day 3 and 5. These metabolites illustrated the increase in lipid metabolism, the perturbation of the pentose phosphate pathway and the TCA cycle in biochip. After drug stimulations, the exometabolome of biochip culture appeared more perturbed than the Petri exometabolome. The GLP1 contributed to the increase in the levels of glycolysis, pentose phosphate and glutathione pathways intermediates, whereas isradipine led to reduced levels of lipids and carbohydrates.

## 1. Introduction

Pancreatic islets or islets of Langerhans are micro-tissues representing approximately 2% of the pancreas and assume its endocrine functions [1]. They harbor distinct cell types including α, β, δ, ε, and γ (PP: pancreatic polypeptides) cells. The main populations are β- and α-cells, which maintain glucose homeostasis within a narrow physiological range through the secretion of insulin and glucagon, respectively [2]. The lack or insufficiency of pancreatic endocrine function resulting from loss of β-cell mass, disrupted insulin secretion and/or impaired insulin action in target tissues leads to a permanent high level of blood glucose (hyperglycaemia) and diabetes mellitus (DM) [3]. The diabetic state is characterized by random plasma glucose concentration, more than 11.1 mmol/L, and a fasting plasma glucose concentration in excess of 7 mmol/L [4].

DM can be categorized into four types: Type 1 DM (T1DM) caused by autoimmune destruction of β-cells, Type 2 DM (T2DM) due to impaired insulin secretion and the insensitivity of target tissues to insulin, gestational diabetes mellitus (GDM), and a fourth category that includes less common types of DM (caused by genetic mutations and drug exposure) [5,6]. T1DM and T2DM are the most common forms and account for 90% to 95% of DM cases [7]. Diabetes affects approximately 463 million people worldwide and is predicted to concern 700 million people by 2045 [8]. The International Diabetes Federation (IDF) and the World Health Organization (WHO) have identified DM as a significant health concern of the 21st century, causing 4.2 million deaths annually, and it is associated with multiple severe complications, such as blindness, kidney failure, cardiovascular disease, sexual dysfunction, neuropathy and peripheral vascular disease [9,10]. Despite the advances in diabetes research, to date, no robust treatment strategies have been developed and the occurrence of DM continues to rise dramatically [11]. As a result, there is a need to develop relevant models and powerful analytic tools to increase the knowledge of the underlying mechanisms of diabetes and to identify new therapeutic solutions and anti-diabetic drugs.

In diabetes research and drug development, most commonly, animal models are employed. Thus, numerous models have been developed in recent decades [12]. However, these models tend to fail due to the differences between humans and animals and the complexity of the disease [5]. On the other hand, the current standard for in vitro testing, i.e., conventional Petri dish culture, does not reproduce the key aspects of in vivo cell microenvironment [13,14]. In recent years, there has been an ongoing effort to propose in vitro models mimicking in vivo cell behavior and environment such as 3D cultures, co-cultures and the perfused microfluidic culture. Therefore, many groups have developed tissue-engineering and 3D culture processes in order to provide a more appropriate environment for pancreatic tissue maintenance and development [14,15,16]. One of the promising approaches is the cell/tissue culture in a dynamic microfluidic device. This technology allows to reconstitute the controlled “physiological-like” micro-environment, the complex function of human organ or a group of organs and the in vivo cellular metabolic responses [17,18]. In this frame, several groups have developed microfluidic biochips to culture and assess pancreatic islet or β-cell spheroids functionalities [11,14,19,20,21,22,23,24,25]. Our group proposed several cell cultures in microfluidic biochips and presented new insight in pancreas metabolism [26,27], liver metabolism [28] and liver–pancreas interaction [29].

In the past decade, the advent of high throughput omics technologies (transcriptomic, proteomic, metabolomic, genomic and lipidomic) has fueled the discovery-based approach by providing access to larger quantitative datasets. Among omics technologies, metabolomic represents a powerful analytical tool to characterize and acknowledge the metabolism of biological systems [30]. Metabolomics, or the study of the metabolome, focuses on the detection and identification of low molecular weight compounds (metabolites < 1500 Da) mainly using nuclear magnetic resonance (NMR) spectroscopy or mass spectrometry (MS) [31,32]. The metabolites are produced by chemical reactions or metabolic process taking place in cells or the whole organism and represent phenotypic end-products (the downstream expression of genome, transcriptome and proteome) of living organisms [33,34]. Their characterization makes it possible to identify variations in the culture medium or the cellular fluid composition and provide the detailed information of the metabolic changes [34]. Metabolomic has been used to study pancreatic islet physiology and pancreatic disorder signatures, as well as to identify early biomarkers [35,36,37]. Our group has also demonstrated the potential of integrating cell culture in microfluidic biochips with metabolomic to identify xenobiotics biomarkers and signatures [38,39].

In this work, we investigate the exometabolome [40,41] of rat islets of Langerhans cultured in Petri and microfluidic biochip when exposed to high-glucose concentration. In addition, we studied the effect of the glucagon-like peptide-1 (GLP-1) and isradipine drugs. GLP-1 physiologically induces glucose-dependent insulin secretion from β-cells and GLP-1 analogues improve hyperglycemia in T2D patients. Isradipine is a calcium antagonist used as an antihypertensive drug in diabetic patients. It was reported to have no effect on glucose tolerance, insulin secretion and insulin action in type II diabetic patients [42]. Nevertheless, it is reported to inhibit the insulin release by blocking the calcium ion channel currents [43,44].

## 2. Materials and Methods

### 2.1. Biochip and Fluidic Circuits

The biochips and the perfusion circuits have been detailed in our previous work [27]. The microfluidic biochip (pancreatic islet biochip) consists of two layers (polydimethylsiloxane PDMS, Sylgard 184 kit, Dow Corning) sealed by reactive air plasma treatment (plasma cleaner, Harrick Plasma). This led to a closed biochip (total volume of 40 µL and 2 cm^2^ of surface) with one inlet and one outlet to perfuse the pancreatic cultures. To trap and keep the islets inside the biochip, the bottom layer is composed of 600 micro-wells of 400 µm in diameter (depth of 300 µm), and spaced 50 µm apart (Figure S1A).

For dynamic culture, the biochips were connected to perfusion circuits composed of silicone/Teflon tubing (0.65 mm in diameter), bubble trap and peristaltic pump. The bubble trap was used as a culture medium reservoir. The entire setup is presented in Figure S1B. Before the experiments, the biochips and the perfusion circuits were sterilized by autoclaving and dried in an oven.

### 2.2. Culture Medium and Reagents

The islets culture medium used in this study was the classic RPMI 1640 medium (Gibco, Waltham, MA, USA; 11 mmol/L of glucose, following Carter et al. 2009 recommendation [45]) supplemented with 10% FBS (Gibco), 100 units/mL of penicillin, 100 mg/mL streptomycin (Gibco) and GlutaMAX™ (Gibco™) at 10 mmol/L. For glucose-stimulated insulin secretion (GSIS) assays, 0-glucose (D-MEM, No Glucose, Fujifilm Wako Chemicals, Osaka, Japan) and high-glucose culture media (D-MEM high Glucose, 25 mmol/L of glucose, Fujifilm Wako Chemicals) were used.

Glucagon-like peptide-1 (GLP-1) and isradipine were purchased from Peprotech (Cranbury, NJ, USA) and Cayman Chemical (Ann Arbor, MC, USA), respectively. Stock solutions of both drugs were prepared in dimethyl sulfoxide (DMSO, Sigma-Aldrich, Saint Quentin Fallavier, France) and RPMI medium for isradipine and GLP-1, respectively. Further dilutions in culture medium were realized to achieve the final concentrations of expositions: 100 nmol/L for GLP-1 and 10 µmol/L for isradipine.

### 2.3. Pancreatic Islet Culture

Pancreatic islets were isolated from male Wistar rats aged 8–9 weeks old (CLEA Japan, Inc., Tokyo, Japan) using the protocol described by Yonekawa et al., 2006 and Kiba et al., 2013 [46,47]. The details of the islet’s extraction protocol are given in our previous work and in the Supplementary File [27]. The rats were housed in ventilated, humidity- and temperature-controlled (22 °C) rooms with a 12 h light/dark cycle, with food and water ad libitum. All animal experimentation procedures were carried out according to the guidelines of the University of Tokyo and the Japanese Ministry of Education.

The biochips and the perfusion circuits were previously filled with culture media in order to remove the air bubbles and moisturize the circuits. The islets in the preservation solution were washed, gently diluted in the appropriate amount of culture medium and ≈40 ± 10 islets/biochip were loaded into the biochips via the inlet ports using a micropipette tip. After 2 h in static conditions, to promote islet sedimentation in the microwells, the circuit was connected to the peristaltic pump and the perfusion started (flow rate of 20 µL/min). The biochips were perfused in a closed-circuit loop containing 2 mL of culture medium. After Day 1, the medium was renewed/collected every 2 days (on Day 3 and Day 5). The entire setup was continuously incubated at 37 °C in a 5% CO_2_ supplied incubator. In static conditions, ≈40 ± 10 islets/well were loaded into 24-well plate and incubated in the same conditions. The volume of culture medium was of 1 mL/well and the medium was exchanged every day.

### 2.4. Islet Viability and Functionality

Cell viability was assessed at the end of the experiment using propidium iodide (PI)/calcein-AM staining. For that purpose, the islets were incubated in a solution of propidium iodide (PI) at 4.5 µmol/L and calcein-AM at 2 µmol/L (Cellstain kit, Dojingo, Kumamoto, Japan) in RPMI 1640 medium for 30 min. Then, the samples were washed with RPMI 1640 medium and observed under an epifluorescence microscope (Olympus, Tokyo, Japan). The cell viability in pancreatic islets was quantified by the ImageJ software (NIH, Bethesda, MD, USA) using the collected images. The area of the cells stained with calcein-AM (living cells) was measured and normalized by the total area of the islets.

The nuclei and F-actin stainings were performed using fixed islets. After washing with phosphate-buffered saline solution (PBS, Gibco), the islets were permeabilized with 1% Triton X100 in PBS for 3 h at 4 °C and washed 3 times with PBS for 30 min. Actin filaments were stained with Phalloidin-iFluor488 Reagent (Abcam, Cambridge, UK) and diluted in the range recommended by the manufacturers (incubation for 3 h at room temperature in the dark). The nuclei were stained with Hoechst 33342 (H342, Dojindo, Kumamoto, Japan) at 1/800 for 30 min at room temperature in the dark. The observations of the stained samples were made with an Olympus IX-81 confocal laser-scanning microscope. 

The insulin, C-peptide and glucagon secretions in the culture medium were assessed using ELISA assays. The used ELISA kits were rat insulin ELISA kit (10-1250-01; Mercodia, Uppsala, Sweden) for insulin, Glucagon DuoSet ELISA kit (DY1249; R&D Systems, Tokyo, Japan) with the DuoSet ELISA Ancillary Reagent Kit 2 (DY008; R&D Systems) for glucagon and rat, and C-Peptide ELISA kit (10-1172-01; Mercodia) for C-peptide. The results were obtained using an iMark microplate reader (Bio-Rad, Osaka, Japan) set to a wavelength of 450 nm. 

To evaluate islet responsiveness to low/high-glucose stimulation, the islets in Petri and biochip were exposed successively to 0-glucose (2 h) and high-glucose (2 h) culture media (detailed protocol in Supplementary File). The GSIS (glucose-stimulated insulin secretion) index was calculated by dividing insulin measured in high-glucose and low-glucose media.

All experiments were repeated at least three times, and the data are presented as the mean ± SD. One-way ANOVA was performed for statistical analysis (GraphPad Prism 8 software, State College, CA, USA). A *p* value less than 0.05 was considered as statistically significant.

### 2.5. Exometabolomic Analysis

The exometabolomic profiling was performed with culture media collected during two days of contact with islets. We analyzed the culture medium collected on Day 3 (Day 1 to 3) and Day 5 (Day 3 to 5). The analysis was performed with samples from at least three independent experiments. Firstly, an extraction step was performed according to the protocol previously described by Jellali et al. (2018) [39]. Then, samples were analyzed by gas chromatography (GC, Agilent 7890B) coupled to quadrupole mass spectrometry (MS, Agilent 5977A). Separation was achieved on a Rxi-5SilMS column from Restek (30 m with a 10 m Integra-Guard column—ref 13623-127). The AMDIS software (National Institute of Standards and Technology, MD, USA) was used for data analysis. The detailed protocols are given in the Supplementary File and in our previous work [48].

The multivariate statistical analysis of exometabolomics data was performed using MetaboAnalyst 5.0 [49]. Supervised partial least squares-discriminant analysis (PLS-DA) and orthogonal projections to latent structures-discriminant analysis (OPLS-DA) were applied to obtain the maximum separation between control and treated groups, and to explore the variables that contributed to this separation. The quality of models was evaluated by the R^2^Y (fitting degree) and Q^2^ (prediction parameter) values. The significant metabolites were selected using variable importance in the projection value (importance of each variable to the whole model, VIP > 1) and *p* value (*p* < 0.05, Student’s *t*-test). Pathways and enrichment analysis were performed with MetaboAnalyst using the selected significant metabolites.

## 3. Results

### 3.1. Morphology and Functionality of Pancreatic Islets in Petri and Biochip

**Figure 1:**
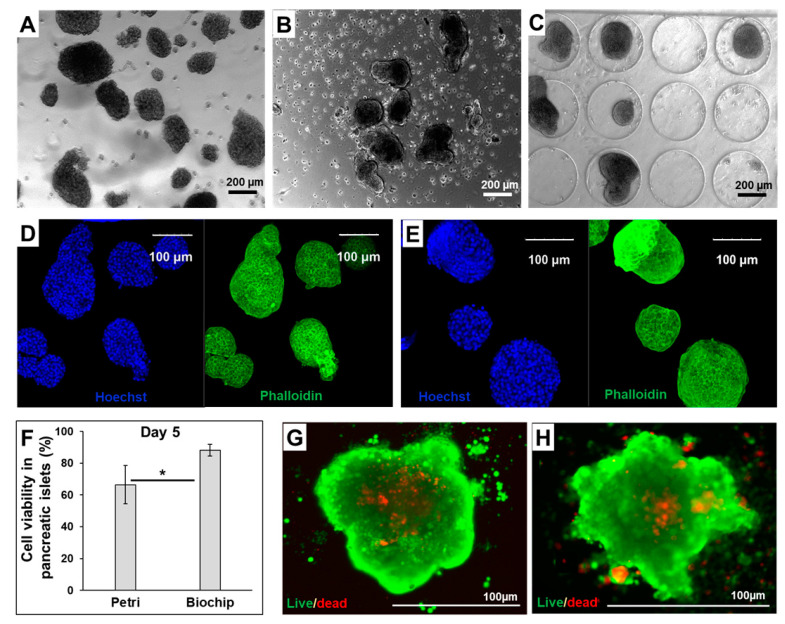
Islet morphology and viability. (**A**–**C**) morphology of the islets after extraction, after 5 days of culture in Petri and after 5 days of culture in biochip, respectively; (**D**,**E**) Hoechst (nuclei, blue) and phalloidin (F-actin, green) staining of islets cultured in Petri and biochip, respectively; (**F**) cell viability in pancreatic islets at the end of the experiment (day 5); (**G**,**H**) live/dead staining at the end of the experiment (day 5) for islets cultured in Petri and biochip, respectively; calcein (living cells, green) and propidium iodide (dead cells, red). * *p* < 0.05.

The pancreatic islet cultures were performed for 5 days in static (Petri) and dynamic (perfused biochip) conditions. The islets’ morphologies after extraction and at the end of cultures in Petri and biochip are presented in Figure 1A, Figure 1B, Figure 1C, respectively. In both Petri and biochip cultures, we confirmed the presence of healthy islets displaying the spherical shape of pancreatic islets. The cytoskeleton organization was investigated by actin staining. As shown in Figure 1D,E, the actin cytoskeleton of the cells can be seen clearly in the whole of each islet. Moreover, at the end of experiment, we collected the same number of islets as those seeded. As shown in Figure 1F–H, the cell viability was higher in the islets cultivated in the biochips (88 ± 3.5%) when compared to Petri situations (67 ± 12%).

**Figure 2:**
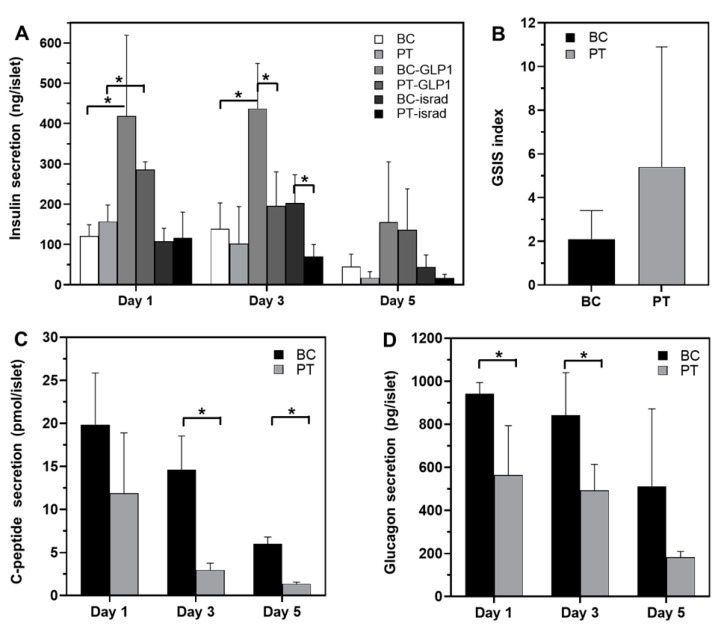
Hormone secretions in biochip (BC) and Petri (PT) throughout the 5 days of culture. (**A**) insulin secretion in control and after exposure to GLP-1 and isradipine (biochip and Petri); (**B**–**D**) glucagon, C-peptide secretions and GSIS index (biochip and Peri control), respectively. GSIS: glucose-stimulated insulin secretion index = insulin measured in high-glucose/insulin measured in low-glucose. * *p* < 0.05.

To evaluate the islets’ functionality, insulin, C-peptide and glucagon secretions were quantified throughout the culture (Figure 2). We found that basal secretions of three hormones were higher in the biochip than in the Petri. However, the kinetics analysis showed that the islets’ secretions decreased over time in both cultures’ modes. On Day 3, the insulin, C-peptide and glucagon secretions were 139 ± 64 ng/islet, 14.64 ± 3.9 pmol/islet and 842 ± 198 pg/islet in biochip and 102 ± 92 ng/islet, 2.97 ± 0.8 pmol/islet and 494 ± 120 pg/islet in Petri, respectively. The glucose-stimulated insulin secretion highlighted also the islets functionality in both culture modes (Figure 2B). Finally, the islets’ response to GPL1 stimulation was confirmed by the increase in the insulin secretions (Figure 2A). Indeed, the levels of insulin in the GLP1-treated Petri and biochips were up to 2–3 times higher than those attained in control. However, in the present experience, we did not detect any effect of isradipine on the insulin secretion (Figure 2A).

### 3.2. Comparison of the Exometabolomes of Islets Cultured in Petri and Biochip

#### 3.2.1. Global Multivariate Analysis

**Figure 3:**
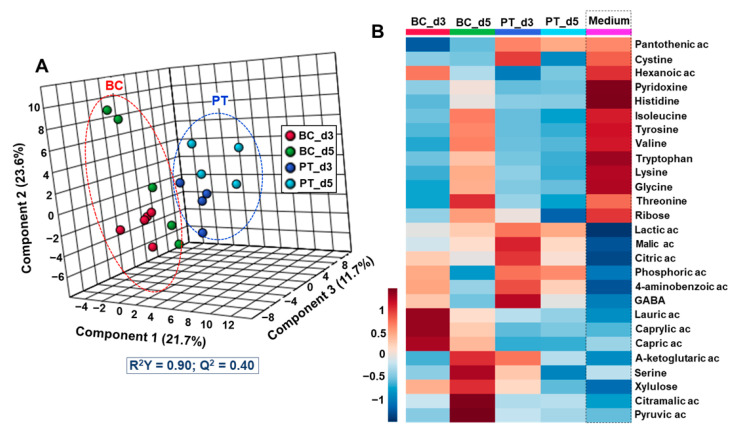
Global multivariate statistical analysis of Petri and biochip metabolomic profiles on Day 3 and 5. (**A**) PLS-DA scores plot discriminating Petri and biochip cultures; (**B**) heatmap of 27 metabolites significantly modulated. PLS-DA: partial least squares-discriminant analysis; BC_d3 and BC_d5: biochip cultures after 3 and 5 days; PT_d3 and PT_d5: Petri cultures after 3 and 5 days.

The exometabolomic analysis led to the identification of 100 different metabolites in the culture medium samples (Table S1). To identify the exometabolomic signature of each culture mode, we performed a multivariate analysis to compare the exometabolome of pancreatic islets, cultured in biochip and Petri, on Day 3 and Day 5 (BC_d3 vs. BC_d5 vs. PT_d3 vs. PT_d5). The PLS-DA score plots showed a clear separation between Petri and biochip cultures, indicating distinct metabolic profiles (Figure 3A). The analysis discriminated also the exometabolomic profiles on Day 3 and Day 5, both in biochip and Petri. An ANOVA test coupled with the PLS-DA analysis contributed to the extraction of 27 metabolites with a *p* value below 0.05 and VIP (variable importance for the projection) scores of >1. The heatmap of the 27 significant metabolites used to discriminate biochip and Petri culture is given in Figure 3B.

The specific signature of the biochips on Day 3 was characterized by the high secretion of lipids, including capric, caprylic and lauric acids. On Day 5, the biochip culture exhibited a high production of pyruvic acid, citramalic acid, xylulose and ketoglutaric acid, whereas amino acid consumption (tryptophan, isoleucine, tyrosine, lysine, glycine, valine, threonine and histidine) was low compared to Day 3. Concerning Petri, the cultures exhibited a high consumption of amino acids (isoleucine, histidine, tyrosine, tryptophan, valine, lysine and glycine), pyridoxine and ribose both on Day 3 and Day 5. Day 3 of Petri cultures was characterized by high secretion of citric, malic, lactic and phosphoric acids, GABA, and cystine. A weak production of these compounds was also observed on Day 5. Finally, when comparing both Petri vs. Biochip, we found that the biochips’ cultures commonly led to a high secretion of capric, caprylic, hexanoic and lauric acids, and xylulose, whereas the Petri cultures commonly led to citric, malic, phosphoric, and lactic acids production.

#### 3.2.2. Differential Analysis of the Petri vs. Biochip Culture Modes

In order to identify the differences in the exometabolomic signature of each culture’s mode throughout the 5 days of the experiment, we refined the analysis by comparing the biochip and Petri cultures at each time point (BC_d3 vs. PT_d3 and BC_d5 vs. PT_d5).

**Figure 4:**
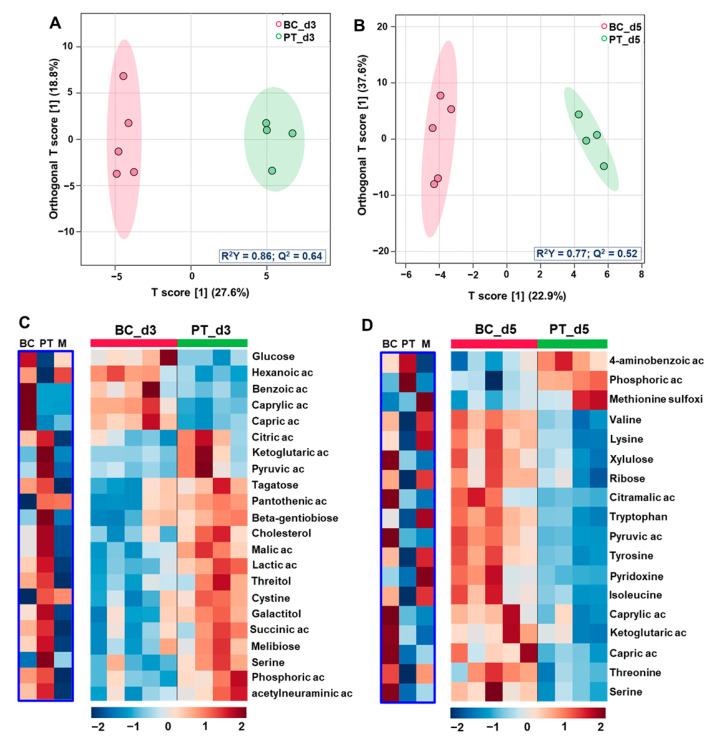
Comparison of metabolomic profiles of biochip and Petri cultures at different time points. (**A**,**B**) OPLS-DA score plots of Petri and biochip cultures comparisons on Day 3 (BC_d3 vs. PT_d3) and Day 5 (BC_d5 vs. PT_d5), respectively; (**C**,**D**) heatmap of metabolites significantly modulated in BC_d3 vs. PT_d3 and BC_d5 vs. PT_d5 comparisons, respectively. The blue box represents the levels of metabolites in Petri (PT) and biochip (BC) compared to the initial culture medium (M). OPLS-DA: orthogonal projections to latent structures-discriminant analysis; BC_d3 and BC_d5: biochip cultures after 3 and 5 days; PT_d3 and PT_d5: Petri cultures after 3 and 5 days.

**Figure 5:**
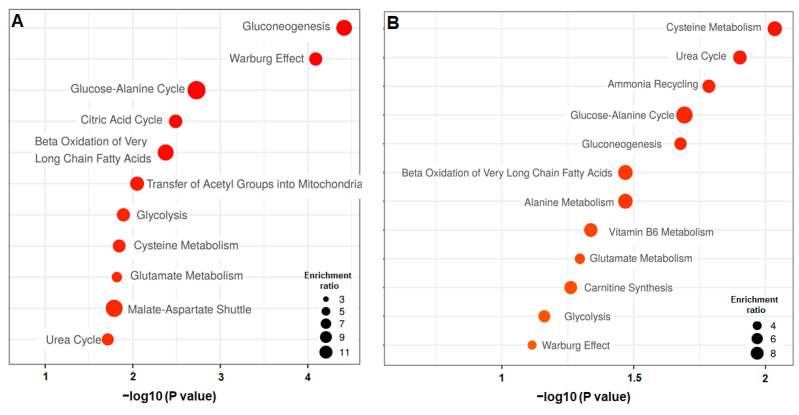
Pathway impact enrichment extracted from the Petri and biochip cultures comparisons. (**A**) biochip culture after 3 days (BC_d3) vs. Petri cultures after 3 days (PT_d3) comparison; (**B**) biochip culture after 5 days (BC_d5) vs. Petri cultures after 5 days (PT_d5) comparison.

OPLS-DA analysis performed with samples of biochip and Petri on Day 3 contributed to distinguishing both modes of culture and to extracting 22 metabolites significantly modulated (*p* value < 0.05). The OPLS-DA score plot and the heatmap of discriminant metabolites are presented in Figure 4A and Figure 4C, respectively. The full list of metabolites (with *p* value and VIP score) is also provided in Table S2. We found that the secretion of benzoic acid and three lipids (hexanoic acid, capric acid and caprylic acid) was higher in biochip, whereas TCA intermediates (citric, pyruvic, malic, lactic and alpha-ketoglutaric acids), several carbohydrates (threitol, melbiose and galactitol), cholesterol, serine, phosphoric acid and n-acetylneurminic acid were particularly produced in Petri cultures. The pathway enrichment with MetaboAnalyst (using the 22 extracted metabolites) led to the extraction of the enrichment of beta-oxidation of the long-chain fatty acid, the TCA cycle, the glucose alanine cycle, the gluconeogenesis and the Warburg effect among the top five biological processes (Figure 5A, hits and *p* value are provided in Table S3).

The comparison of the Petri and biochip cultures on Day 5 led to a statistical difference as illustrated by the two distinct clusters formed in the OPLS-DA score plot (Figure 4B). In total, 18 metabolites were significantly different (*p* value < 0.05 and VIP > 1) between the BC_d5 and PT_d5 conditions (the heatmap is provided in Figure 4D and the full list is provided in Table S2). The metabolites list included high levels (secretion) of phosphoric acid and 4-aminobenzoic acid in Petri. In Biochips, we detected high levels of amino acids, suggesting a weak consumption: valine, tryptophan, tyrosine, serine, threonine and isoleucine. A high secretion of carbohydrates (ribose, xylulose), TCA intermediate (pyruvic, citramalic and alpha-ketoglutaric acids) and lipids (capric and caprylic acids) was also detected. The resulting pathway enrichment with those top 18 metabolites included ammonia recycling, urea cycle, cysteine metabolism, beta-oxidation of long-chain fatty acid, TCA cycle, glucose alanine cycle, gluconeogenesis among the top six enriched pathways (Figure 5B, hits and *p* values are provided in Table S3).

#### 3.2.3. Differential Analysis between the Day 3 vs. Day 5

**Figure 6:**
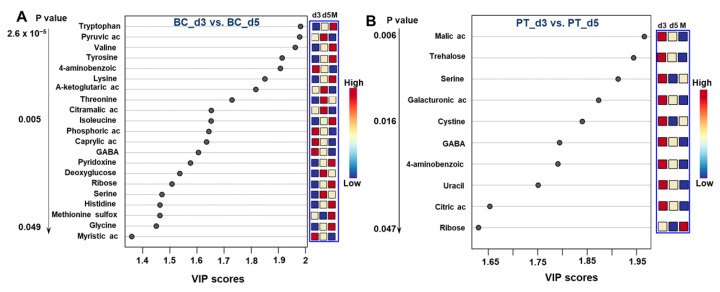
Metabolites discriminating on Days 3 and 5 in both culture modes. Conditions based on VIP scores (variable importance in projection) from OPLS-DA and the corresponding detection intensity measured on each day of culture: (**A**) biochip culture and (**B**) Petri culture. The blue box represents the levels of metabolites in Petri (PT) and biochip (BC) on Days 3 and 5 compared to the initial culture medium (M). OPLS-DA: orthogonal projections to latent structures-discriminant analysis; BC_d3 and BC_d5: biochip cultures after 3 and 5 days; PT_d3 and PT_d5: Petri cultures after 3 and 5 days.

To investigate the kinetics of the metabolites throughout islet culture, we analyzed and compared the temporal evolution of the metabolites in each culture mode separately. In biochips, Day 3 and Day 5 were successfully separated as two distinct clusters were formed in OPLS-DA analysis (OPLS-DA score plot in Figure S2A). We extracted 21 metabolites discriminating the exometabolomic profiles of Day 3 and Day 5 (with a *p* value < 0.05 and VIP > 1). The expression levels of the 21 metabolites with the highest VIP scores separating biochip cultures on Day 3 and 5 and the corresponding heatmap are shown in Figure 6A and Figure S2C, respectively (full list of metabolites presented in Table S2). Day 3 was characterized by the high levels (secretion) of caprylic and myristic acids, GABA, phosphoric acid and 4-amninobenzoic acid. The production of those metabolites decreased between Day 3 and Day 5. On day 5, we particularly monitored a low consumption (compared to Day 3) of amino acid and the production of pyruvic, alpha-ketoglutaric and citramalic acids (VIP score above 1.7). The enrichment analysis performed with those 21 metabolites highlighted the ammonia recycling pathway, the glutamate, alanine and cysteine metabolisms, as well as the carnitine synthesis (Table S3).

In Petri, we discriminated only 10 metabolites (with a *p* value below 0.05) differentially expressed between Days 3 and 5. All of these metabolites (citric acid, malic acid, uracil, trehalose, galacturonic acid, GABA, 4-aminobenzoic acid, serine and cystine) were higher on Day 3 (production), when compared to Day 5 (Figure 6B). In particular, serine and cystine were produced on Day 3 and consumed on Day 5 (in comparison with the initial culture medium). The heatmap and full analysis are presented in Figure S2B, S2D and Table S2, respectively. The enrichment analysis using Metaboanalyst did not lead to significant enrichment (Table S3).

### 3.3. Effect of GLP1 Treatment on the Pancreas Metabolome

The pancreatic islets cultured in Petri and biochip were exposed to chronic GLP1 treatment. The metabolic profiling was performed on Days 3 and 5 and compared to islets cultures without treatment (control). After 3 days of culture, GLP1 treatment weakly perturbated the exometabolome of pancreatic islets (results not shown).

**Figure 7:**
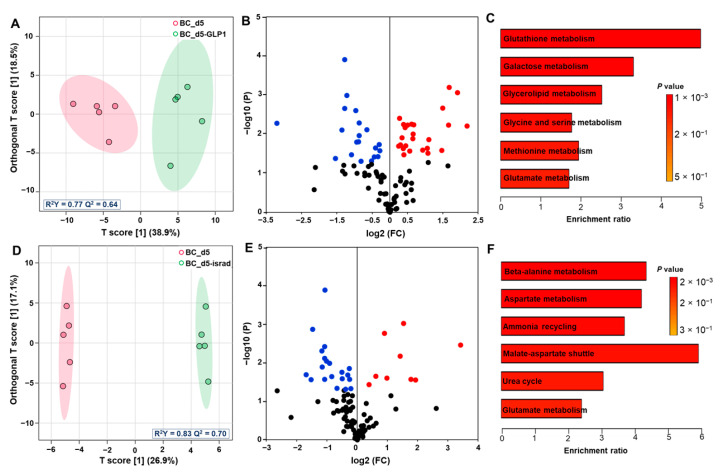
Comparison of metabolomic profiles of biochip control culture and biochip culture treated with GLP1 and isradipine. (**A**,**D**) OPLS-DA score plot of biochip control culture compared to biochip treated with GLP1 and isradipine on Day 5, respectively; (**B**,**E**) volcano plot (log2 fold change (treated biochip/control biochip) plotted against −log10 *p*-value) highlighting metabolites differentially expressed between control biochip and biochip treated with GLP1 and isradipine, respectively. Metabolites upregulated and downregulated in the treated cultures are labelled in red and blue, respectively; (**C**,**F**) pathway impact enrichment based on metabolites discriminating biochip control and biochip treated with GLP1 and isradipine, respectively. OPLS-DA: orthogonal projections to latent structures-discriminant analysis; BC_d5: biochip cultures after 5 days; BC_d5-GLP1: biochip culture treated with GLP1 after 5 days; BC_d5-israd: biochip culture treated with isradipine after 5 days; GLP1: glucagon-like peptide-1; israd: isradipine.

After 5 days of culture in biochip, the GLP1 treatment led to an intense exometabolome perturbation. The OPLS-DA analysis allowed us to separate islet control cultures and islets exposed to GLP1, as shown in Figure 7A. The models fitted the data well and showed good predictability (R^2^ = 0.77, Q^2^ = 0.64). To extract the modulated metabolites, a volcano plot was plotted using the *p* value (-log10P) and fold change (log2FC). The metabolites extracted were also validated by a VIP score (VIP > 1). When compared to control, the GLP1 treatment led to the modulation of 45 metabolites (Figure 7B and Table 1). The heatmap of the 45 metabolites and the full analysis (all metabolites with *p* value, VIP and FC) are also provided in Figure S3A and Table S4, respectively. The most significantly modulated metabolites by GLP1 were amino acids (isoleucine, valine, tyrosine and methionine) that were detected at high level (compared to culture without GLP1), indicating low consumption. In parallel, the production of fructose, myoinositol, xylulose, pyroglutamic acid, proline and creatinine increased after GLP1 treatment. Finally, GLP1 treatment led to a decrease in the secretion of several metabolites, such as arabinose, melibiose, mannose, trehalose, glycerol-1-phosphate, glyceric acid and kynurenine. The pathway enrichment analysis with the top 45 metabolites highlighted the glutathione, galactose (carbohydrates-related pathway) and glycerolipid metabolisms (Figure 7C, the full list of the pathways is given in Table S5).

In Petri on Day 5 (with GLP1 treatment), we discriminated 11 metabolites with a *p* value below 0.05 increase in hypoxanthine, threitol, pyruvic acid, tagatose, fructose and beta-hydroxyvalerate production and decrease in tryptophan and tyrosine consumption (Table 1 and Table S4, Figure S4A). The enrichment analysis with those top 11 metabolites led to the extraction of mainly the amino sugar metabolism and glucose-alanine cycle (Table S5).

### 3.4. Effect of Isradipine Treatment on Pancreas Exometabolome

The exometabolomic profiling was also performed for pancreatic islets cultivated in biochip and Petri, and exposed to isradipine. As for the treatment with GLP1, both in Petri and biochip, isradipine treatment weakly perturbated the exometabolome of pancreatic islets on Day 3. Therefore, we focused on the metabolic profile after 5 days only.

In biochip, we successfully separated the profile of control and islets treated with isradipine using OPLS-DA analysis (R^2^Y = 0.83 Q^2^ = 0.70, Figure 7D). The volcano plot in Figure 7E allowed the extraction of 30 metabolites modulated by isradipine treatment (*p* value < 0.05, Table 1 and Table S4, Figure S3B). Among them, we found a decrease in the production of several carbohydrates (mannose, galacturonic acid and sorbitol) and lipids (palmitic, oleic, stearic and arachidic acid) in the isradipine culture when compared to controls. In parallel, a reduced consumption of amino acid was highlighted by the high levels of methionine, proline, valine, cysteine, asparagine and glutamine in the culture treated with isradipine. The loading of those metabolites in Metaboanalyst (enrichment analysis) contributed to the extraction of significant perturbation (with high impact and low *p* value) of the beta-alanine metabolism, aspartate metabolism, ammonia recycling, malate-aspartate shuttle and glutamate metabolism (Figure 7F, Table S5).

The comparison between Petri culture on Day 5 (control vs. isradipine) highlighted only two metabolites with a *p* value below 0.05 (Table 1) and seven metabolites with a *p* value below 0.1 (Figure S4B and Table S4). The treatment reduced the production of benzoic acid, galactitol, arabinose and eicosapentaenoic acid, and increased alanine and glycerol secretion. The pathway enrichment analysis with the seven metabolites extracted only the galactose metabolism (*p* value = 0.025, Table S5).

## 4. Discussion

Biomechanical and biochemical stimulations, such as shear stress, cell interactions and soluble factors concentrations, largely impact pancreatic islet function and responses [50]. In vivo, pancreatic islets are exposed to a flow rate estimated at 0,03 mL/day [20]. In our biochip, the dynamic cultures were performed at 20 µL/min. Using Hele Shaw chamber approximation [51], we estimated the velocity, shear stress and Reynolds number (Re) equal to 83 µm/s, 0.00125 Pa and 3.3, respectively. Although the flow rate is different from that estimated by Sankar et al., the shear stress of 1.25 mPa in our biochip remains within the range of values beneficial for pancreatic in vitro cultures in microfluidic biochip [23,52]. Furthermore, the selection of the flow rate is based on our previous work demonstrating healthy and functional pancreatic cultures in microfluidic biochip (for both rat islets and iPSCs-derived β-cell spheroids) [26,27,29]. Particularly, the culture of rat islets in our microfluidic biochip maintained high viability and functionality, and led to an overexpression of several important pancreatic islet genes (in comparison with static culture in Petri) [27]. Pathological exposure to prolonged high-glucose concentration, simulating glucotoxicity, is reported to induce β-cells apoptosis [53], oxidative stress [54], reduction in insulin gene expression [55] and to alter glucose metabolism [56]. At the metabolome level, in the literature, it was characterized by high fatty acid productions, increased levels of pentose phosphate pathway intermediates and glycolysis intermediates. It is also reported that the levels of GABA, hydroxyproline and glycine were increased, whereas nitrogen-related metabolites, such as glutamine, serine, ornithine, aspartate were reduced [36]. In our experiment, the level of glucose remained high, close to 10 mmol/L (Figure S5), simulating continuous high-glucose exposure (both in Petri and biochips). Our biochip data consistently highlighted the increase in lipid metabolism (especially on Day 3 via a high production of caprylic, capric and lauric acids coupled with a consumption of hexanoic acid, see Figure 3B, and a low production of cholesterol, see Figure 4C). We also confirmed the consumption of ribose and a higher production of xylulose between Days 3 and 5, illustrating the perturbation of the pentose phosphate pathway, an increase in the production of metabolites in the TCA cycle (lactic, malic, citramalic and alpha-ketoglutaric acids), in the nitrogen cycle (serine) and in the glycolysis metabolism (pyruvic acid), consistent with the literature reports. Our findings in biochip could, therefore, reflect a glucotoxicity-like signature, consistent with the description of Gooding et al., 2016 [36].

The specific comparison of the pancreatic signatures in biochip and Petri demonstrated several important differences in the nitrogen, lipid and carbohydrate metabolisms. We detected a higher production of cholesterol in Petri on Day 3. Cholesterol is mainly produced in the liver but can be also be produced in pancreatic cancer. It is reported as a key component of the cell membrane during tumor proliferation (abnormal lipid production, such as phospholipids and glycolipids, also occurred during cancer progression) [57,58]. Under hypoxia, cholesterol and lipids are produced via glutamine degradation (canonical pathway in mitochondria and non-canonical pathway in cytoplasm) leading to a sequential formation of alpha-ketoglutaric, isocitric and citric acids, and then, acetyl CoA [59]. In our experiments, the cholesterol production on Day 3 was consistent with the high level of TCA cycle intermediates in Petri, such as citric, malic, lactic, succinic and alpha-ketoglutaric acids, illustrating the intense TCA activation. In parallel, we did not detect high cholesterol production in biochips, but we measured a higher secretion of medium-chain triglycerides (MCT), such as caprylic and capric acids (on Day 3 and Day 5). We did not identify the sources of those MCT. However, MCT are reported to reduce insulin resistance [60] and hepatic cholesterol [61], and to be beneficial against obesity [62]. MCTs are also involved in the modulation of urea synthesis and glucose production [63]. The literature reports are consistent with our findings as far as, concomitantly to the increased of lipids, we also detected higher insulin secretion in biochips, modulation of the nitrogen pathways, glucose alanine cycle, gluconeogenesis on Day 5 when compared Petri and biochip (Figure 5B).

The pancreatic islets cultivated in Petri were poorly responsive to drugs, whereas an intense exometabolome perturbation in response to GLP1 and isradipine were observed for islets cultivated in biochips. The GLP1 normally simulates the hyperglycemic state by stimulation of glucose-dependent insulin secretion. Physiological GLP1 production is reduced in TD2M patients. That is the reason why it is a potential therapeutic target [64]. The literature reported the perturbation of the metabolome in hyperglycemic patients resulting from glucose, arginine and GLP1 stimulations [65]. This glucose stimulation led to the reduction in amino acids, sphingolipids, biogenic amines, phosphatidyl-cholines levels in blood. Furthermore, GLP1 treatments also contributed to the increase in the depletion of several of those metabolites (acylcarnitines, amino acids, biogenic amines and phosphatidylcholines) [65]. Another metabolomic analysis of healthy human pancreatic β-cell islets in response to hyperglycemic stimulation (glucose-stimulated insulin secretion) extracted over expression of metabolites involved in the glycolysis (lactate, pyruvate, fructose-phosphate and fructose bi-phosphate), pentose phosphate pathway (ribose-5 phosphate) and TCA cycle (fumarate, succinate, malate and alpha-ketoglutarate) [36]. In our investigation, the GLP1 treatment contributed to the increase in the insulin secretion and the modulation of islet exometabolome. Consistently with the literature, investigating the hyperglycemic state, we detected an increase in the concentrations of several metabolites in the pentose phosphate pathway and glycolysis (fructose, xylulose and ribose), especially in biochips that appeared more sensitive than Petri cultures. In addition, we found higher productions of proline, serine and glutamine (nitrogen-related amino acids), and lower consumptions of valine, methionine, tyrosine, isoleucine, leucine, glycine and lysine in the GLP1-treated samples (leading, overall, to an increase in the concentration of amino acids in GLP1-treated biochip medium when compared to the control biochip medium). In parallel, we detected high productions of pyroglutamate (intermediate in the glutathione secretion) and an important glutathione pathway perturbation (Figure 7 and Figure S3). In fact, a similar type of signature (high levels of amino acids due to the reduction in their consumption, TCA perturbation, pyroglutamate and fructose releases) was previously associated with liver cell toxicity and mitochondrial perturbation in biochips [38]. High levels of glucose (10 mmol/L) coupled with GLP1 might contribute to a stronger glucotoxicity due to the intense stimulation for the requirement of insulin secretion. In turn, it would lead to islet damages up to a probable important pro-survival metabolic switch.

In the physiological situation, during the glucose-stimulated insulin secretion, the glucose led to the increase of the ATP/ADP ratio, which, in turn, activated Ca2+ and triggered insulin release via Ca2+ signaling [66]. Isradipine is reported to inhibit the calcium current in human pancreatic β-cells and, thus, to contribute to a reduced insulin release [43]. In our dataset, the effect of isradipine was not clearly established on the insulin secretion both in Petri and biochips. This is probably due to the fact that in our protocol the isradipine was supplemented in a high-glucose medium (nb. our configuration can reproduce a situation mimicking an antihypertensive drug effect on metabolic control in diabetic patients). Several reports noticed that this configuration can lead to any isradipine effect on insulin secretion [42]. However, in biochips, isradipine increased the production of nicotinic acid. Nicotinic acid is a precursor of NAD+ that is a cofactor of oxydo-reduction in lipids and carbohydrate metabolisms. The lipids’ and carbohydrates’ perturbations in isradipine-treated biochips were illustrated by the decrease in oleic acid, eicosapentaneoic acid, mannose and sorbitol production, and the switch from production to consumption of stearic, palmitic and arachidic acids. Furthermore, as NAD+ is involved in metabolic adaptations leading to higher insulin sensitivity and mitochondrial biogenesis [67], this may be consistent with the absence of reduced insulin secretion in our data.

Finally, we could not find specific metabolomic data in the literature describing the signature of the isradipine action on β-cells. However, calcium antagonist amlodipine is characterized by the decrease in acylcarnitines levels in plasma. Calcium agonist nifedipine is also reported to reduce the intracellular levels of glycerophosphocholine and to slightly increase in pyroglutamic acid in the GSH pathway in renal cells [68]. These data appeared consistent with the modulation of the fatty acid metabolism in our experience (we observed any production of lipids under isradipine treatment) and a moderate GSH pathway perturbation extracted by the pathway enrichment analysis (Figure 7F) on Day 5, in the biochips exposed to isradipine. No trend was detected in Petri. Once again, the biochip cultures appeared more perturbated and closer to the literature data.

Although we investigated several physio-pathological-like features, our model still presented some limitations. In the present experiments, we limited our characterization to up to 5 days. As diabetes is a chronic disorder, longer time analysis would be required to be able to extrapolate. Furthermore, the analysis was performed with a rat model that could limit the translation to human health. Finally, the pancreas is normally in interaction with other organs, such as the liver, to regulate the glycemia. Nevertheless, we are working on those current issues and we believe that our technology is a step forward in the knowledge of the fight against diabetes.

## 5. Conclusions

In this work, we have compared the exometabolomic profile of rat pancreatic islets of Langerhans cultivated in conventional Petri and microfluidic biochip. The microfluidic culture modified the exometabolomic profiles of the islets when compared to Petri culture. Under high-glucose stimulation, we found that microfluidic biochip culture stimulates mainly medium-chain triglyceride production (capric and caprylic acids) and reduces cholesterol production when compared to Petri situations. In biochips, this was associated with a high production of pyruvate, TCA substrates and with a reduction in several amino acid consumptions when compared to basal medium. Then, we observed that GLP1 and isradipine drug stimulations led to a significant perturbation of the exometabolome in microfluidic biochip cultures. The GLP1 contributed to the increased production of PPP intermediates and glycolysis intermediates and the reduction in amino acid consumptions. The isradipine reduced the lipid productions, especially in biochips and increased the nicotinic acid levels in both Petri and biochips cultures.

## Figures and Tables

**Table 1:** Metabolites significantly modulated by GLP1 and isradipine in Petri and biochip cultures (underline and italics denote metabolites upregulated and downregulated by GLP1 and isradipine treatments, respectively).

	Metabolites
BC_d5-GLP1 vs. BC_d5	*Trehalose, galactonic ac, Beta-gentiobiose, n-acetylneuraminic acid, galactitol, melibiose, cysteinylglycine, trans-4-hydroxyproline, methionine sulfoxide, trans-13-octadecenoic acid, glycerol 1-phosphate, glucosaminic acid, oleic acid, kynurenine, hippuric acid, xanthine, mannose (gluconic ac lactone), arabinose, glyceric acid, 4-aminobenzoic acid* Fructose, myo-inositol, serine, tryptophan, ribose, isoleucine, lysine, tyrosine, norvaline, xylulose, histidine, L-pyroglutamic acid, glycine, glutamine, pyridoxine, creatinine, proline, oxalic acid, alanine, leucine, methionine, valine, glycerol, glutaric acid, cysteine
PT_d5-GLP1 vs. PT_d5	Threitol, hypoxanthine, tagatose, citramalic acid, serine, fructose, pyridoxine, Beta-hydroxyisovalerate, tyrosine, tryptophan, pyruvic ac
BC_d5-israd vs. BC_d5	*Uridine, arachidic acid, trans-4-hydroxyproline, aspartic acid, oleic acid, stearic acid, phenylalanine, mannose (gluconic ac lactone), Beta-alanine, trans-13-octadecenoic acid, palmitic acid, eicosapentaenoic acid, pantothenic acid, kynurenine, xanthine, galacturonic acid, sorbitol, citric acid, glyceric acid, uracil* Glutamine, glutamic acid, 2-phenylacetamide, asparagine, oxalic acid, methionine, valine, proline, cysteine, nicotinic acid
PT_d5-israd vs. PT_d5	*Benzoic ac* Glutaric ac

BC_d5: biochip cultures after 5 days; BC_d5-GLP1: biochip culture treated with GLP1 after 5 days; BC_d5-israd: biochip culture treated with isradipine after 5 days; PT_d5: Petri cultures after 5 days; PT_d5-GLP1: Petri culture treated with GLP1 after 5 days; PT_d5-israd: Petri culture treated with isradipine after 5 days; GLP1: glucagon-like peptide-1; israd: isradipine.

## Data Availability

All data are provided in paper and Supplementary Files.

## Supplementary Materials

The following supporting information can be downloaded at: https://www.mdpi.com/article/10.3390/metabo12121270/s1, Figure S1: (A) design of the biochip used for pancreatic islets culture; (B) setup used for dynamic culture in biochip; Figure S2: comparison of days 3 and 5 in each culture modes. (A, B) OPLS-DA score plots of day 3 and day 5 comparisons in both modes of culture, (BC_d3 vs. BC_d5) and (PT_d3 vs. PT_d5), respectively; (C and D) heatmap of metabolites significantly modulated in BC_d3 vs. BC_d5 and PT_d3 vs. PT_d5 comparisons, respectively. OPLS-DA: orthogonal projections to latent structures-discriminant analysis; BC_d3 and BC_d5: biochip cultures after 3 and 5 days; PT_d3 and PT_d5: Petri cultures after 3 and 5 days; Figure S3: Heatmap of metabolites significantly modulated by GLP1 (A) and isradipine (B) treatments in biochip; Figure S4: heatmap of metabolites significantly modulated by GLP1 (A) and isradipine (B) treatments in Petri. PT_d5: Petri cultures after 5 days; PT_d5-GLP1: Petri culture treated with GLP1 after 5 days; PT_d5-israd: Petri culture treated with isradipine after 5 days; GLP1: glucagon-like peptide-1; israd: isradipine; Figure S5: glucose concentration in Petri and biochip at day 3 and 5 of culture; Table S1: set of metabolites identified in the samples by GC-MS; Table S2: raw data of statistical analysis biochip vs. Petri control; Table S3: raw data of enrichment analysis biochip vs. Petri control; Table S4: raw data of statistical analysis drugs exposure; Table S5: raw data of enrichment analysis drugs exposure. References [69,70] are cited in the supplementary materials.
