# Supplementary material for: Investigation of the Exometabolomic Profiles of Rat Islets of Langerhans Cultured in Microfluidic Biochip
Source: Metabolites. 2022 Dec 15;12(12):1270. doi: 10.3390/metabo12121270 (PMC9786643; doi:10.3390/metabo12121270)
Supplement: Supplementary file 1 [file metabolites-12-01270-s001.zip › Supplementary experimental procedures.docx]

Article

Investigation of the exometabolomic profiles of rat islets of Langerhans cultured in microfluidic biochip

Amal Essaouiba ^1,2^, Rachid Jellali ^1^, Françoise Gilard ^3^, Bertrand Gakière ^3^, Teru Okitsu ^4^, Cécile Legallais ^1^, Yasuyuki Sakai ^2,4,5^, Eric Leclerc ^1,2^*

^1^ Université de technologie de Compiègne, CNRS, Biomechanics and Bioengineering, Centre de recherche Royallieu CS 60319, 60203 Compiègne Cedex, France

^2^ CNRS IRL 2820, Laboratory for Integrated Micro Mechatronic Systems, Institute of Industrial Science,
University of Tokyo, 4-6-1 Komaba, Meguro-ku, Tokyo, Japan

^3^ Institute of Plant Sciences Paris-Saclay (IPS2), UMR 9213/UMR1403, CNRS, INRA, Université Paris-Sud, Université d’Evry, Université Paris-Diderot, Université Paris Saclay, Bâtiment 630 Rue Noetzlin, 91192,
Gif-sur-Yvette Cedex, France

^4^ Institute of Industrial Science, University of Tokyo, 4-6-1 Komaba, Meguro-ku, Tokyo, Japan

^5^ Department of Chemical Engineering, Faculty of Engineering, University of Tokyo, 7-3-1 Hongo,
Bunkyo-ku, Tokyo, Japan

***** Correspondence: eleclerc@iis.u-tokyo.ac.jp

**Supplementary experimental procedures**

**1. Pancreatic islets isolation**

Islets of Langerhans were isolated from male Wistar rats (8–9 weeks old, 200–300 g) (CLEA Japan, Inc, Tokyo, Japan) following a slight modification to the protocol described by Yonekawa et al. (2006) and Kiba et al. (2013). All animal experimentation procedures were carried out in accordance with the guidelines of the University of Tokyo and the Japanese Ministry of Education.

The rats were anesthetized with isoflurane inhalation solution (Pfizer). After clamping of all irrigation blood vessels, the enzymatic solution (Liberase™ TL by Roche) was injected through the bile duct, previously identified and clamped. After the pancreatectomy, there was selective chemical digestion of the organ at 37°C for 30 min with Liberase TL/ ET-K solution (ET‐Kyoto solution, Otsuka Pharmaceutical). The digestion was followed by washing and purification steps using a discontinuous OptiPrep® (Sigma-Aldrich) density gradient. The islets of Langerhans were then identified, individually hand-picked with a Pasteur pipette under a stereomicroscope (Leica S9 D), and transferred to a cold preservation solution made of UW solution (University of Wisconsin, Kneteman et al., 1990) complemented with Miraclid, (Mochika pharmacy, Japan) and heparin, (Mochika pharmacy, Japan). After assessing and counting the islets, the tissue was stored at 4 °C until starting the culture in order to maintain full functional properties as shown in Kimura et al. (2013).

**2.** [**Glucose-stimulated insulin secretion**](https://www.sciencedirect.com/science/article/pii/S0003986108002014) **(GSIS) assays**

In biochips, the culture medium was removed from the bubble trap and the circuit (biochip, tubing and bubble trap) was washed with a 0-glucose solution (D-MEM, No Glucose, Wako) for 2 hours. This washing 0-glucose solution was removed from the bubble trap, and 1 mL of fresh 0-glucose was added and perfused for 2 hours. After this low glucose perfusion, the islets were exposed to a high glucose culture medium for 2 hours (D-MEM high Glucose, Wako, 25 mM of glucose). For that purpose, the low glucose solution was removed from the bubble trap and replaced with 1 mL of high glucose. In a Petri dish, this protocol led to 2h of 0-glucose exposure, followed by another 2h of 0-glucose exposure and finally 2h of high glucose stimulation. At the end of the assays, basal media were re-established for all conditions.

**3. Metabolomic analysis**

The collected culture medium (250 µL) was completed with 500 µL of frozen solution (-20°C) of water:acetonitrile:isopropanol (2:3:3) containing 4 mg/L of adonitol, 2.75 mg/L of α-aminobutyric acid solution (αABA), and placed in an eppendorf thermomixer for 10 min at 4°C with shaking at 1500 rpm. Insoluble material was removed by two centrifugations steps at 14000 rpm for 10 min. Three aliquots of each extract (200 µL) were dried for 4 h at 35 °C in a speed-vac and stored at -80°C until analysis.

For GC-MS injection, samples were taken out of -80°C, warmed for 15 min and dried again for 1.5 h at 35 °C before adding 10 µL of 20 mg/mL methoxyamine in pyridine and the reaction was performed for 90 min at 30 °C with shaking. Then, 90 µL of N-methyl-N-trimethylsilyl-trifluoroacetamide (MSTFA, Regis Technologies) was added and the reaction continued for 30 min at 37 °C. After cooling, 100 µL was transferred to an Agilent vial for injection. Four hours after derivatization, 1 µL of sample was injected in splitless mode on an Agilent 7890B gas chromatograph coupled to an Agilent 5977A quadrupole mass spectrometer. The column was a Rxi-5SilMS from Restek (30 m with 10 m Integra-Guard column - ref 13623-127). An injection in split mode with a ratio of 1:30 was systematically performed for saturated compound quantification. The oven temperature ramp was 60 °C for 1 min then 10 °C/min to 325 °C for 10 min. Helium constant flow was 1.1 mL/min. Temperatures were the following: injector: 250 °C, transfer line: 290 °C, source: 230 °C and quadrupole 150 °C. The quadrupole mass spectrometer was switched on after a 5.90 min solvent delay time, scanning from 50-600 u. Samples were randomized and fatty acid methyl ester mix (C8, C9, C10, C12, C14, C16, C18, C20, C22, C24, C26, C28, C30) was injected in the middle of the queue for external RI calibration.

Raw Agilent datafiles were analyzed with AMDIS (http://chemdata.nist.gov/mass- spc/amdis/). The Agilent Fiehn GC/MS Metabolomics RTL Library (version June 2008) was employed for metabolite identifications. Peak areas were determined with the Masshunter Quantitative Analysis (Agilent) in splitless and split 30 modes. Because automated peak integration was occasionally erroneous, integration was verified manually for each compound and peak areas were normalized to ribitol. Metabolite contents are expressed in arbitrary units (semi-quantitative determination).

**References**

Kiba, T., Tanemura, M., Yagyu, K., 2013. High‐quality RNA extraction from rat pancreatic islet. Cell Biol. Int. Rep. 20, 1-4.

Kimura, Y., Okitsu, T., Xibao, L., Teramae, H., Okonogi, A., Toyoda, K., Uemoto, S., Fukushima, M., 2013. Improved hypothermic short-term storage of isolated mouse islets by adding serum to preservation solutions. Islets 5, 45-52.

Kneteman, N.M., Warnock, G.L., Evans, M.G., Dawidson, I., Rajotte, R.V., 1990. Islet isolation from human pancreas stored in UW solution for 6 to 26 hours. Transplant. Proc. 22, 763-764.

Yonekawa, Y., Okitsu, T., Wake, K., Iwanaga, Y., Noguchi, H., Nagata, H., Liu, X., Kobayashi, N., Matsumoto, S., 2006. A new mouse model for intraportal islet transplantation with limited hepatic lobe as a graft site. Transplantation 82, 712-715.
