# Supplementary material for: Investigation of the Exometabolomic Profiles of Rat Islets of Langerhans Cultured in Microfluidic Biochip
Source: Metabolites. 2022 Dec 15;12(12):1270. doi: 10.3390/metabo12121270 (PMC9786643; doi:10.3390/metabo12121270)
Supplement: Supplementary file 1 [file metabolites-12-01270-s001.zip › Supplementary figures-table.docx]

Article

Investigation of the exometabolomic profiles of rat islets of Langerhans cultured in microfluidic biochip

Amal Essaouiba ^1,2^, Rachid Jellali ^1^, Françoise Gilard ^3^, Bertrand Gakière ^3^, Teru Okitsu ^4^, Cécile Legallais ^1^, Yasuyuki Sakai ^2,4,5^, Eric Leclerc ^1,2^*

^1^ Université de technologie de Compiègne, CNRS, Biomechanics and Bioengineering, Centre de recherche Royallieu CS 60319, 60203 Compiègne Cedex, France

^2^ CNRS IRL 2820, Laboratory for Integrated Micro Mechatronic Systems, Institute of Industrial Science,
University of Tokyo, 4-6-1 Komaba, Meguro-ku, Tokyo, Japan

^3^ Institute of Plant Sciences Paris-Saclay (IPS2), UMR 9213/UMR1403, CNRS, INRA, Université Paris-Sud, Université d’Evry, Université Paris-Diderot, Université Paris Saclay, Bâtiment 630 Rue Noetzlin, 91192,
Gif-sur-Yvette Cedex, France

^4^ Institute of Industrial Science, University of Tokyo, 4-6-1 Komaba, Meguro-ku, Tokyo, Japan

^5^ Department of Chemical Engineering, Faculty of Engineering, University of Tokyo, 7-3-1 Hongo,
Bunkyo-ku, Tokyo, Japan

***** Correspondence: eleclerc@iis.u-tokyo.ac.jp

**Supplementary figures**


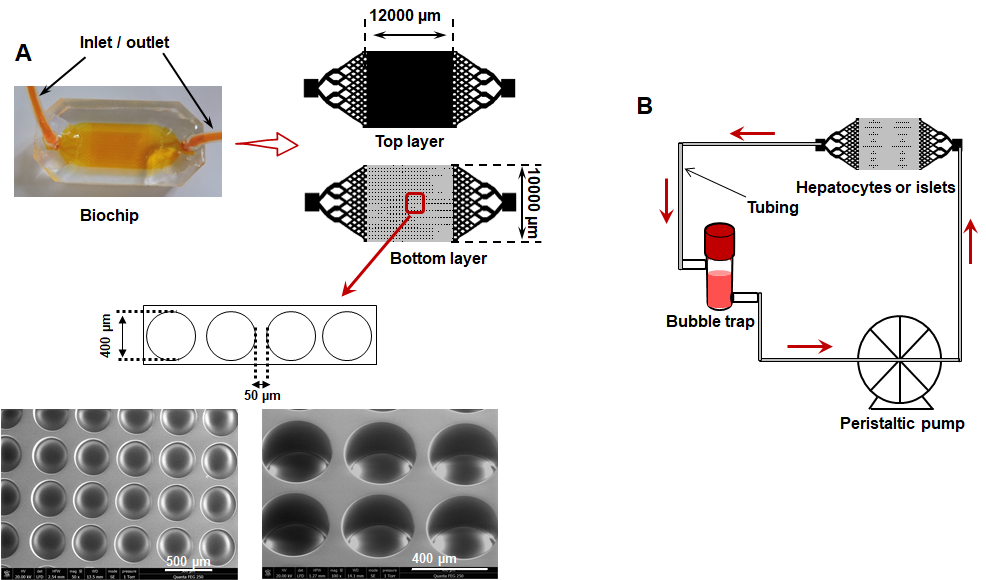


**Figure S1.** (A) Design of the biochip used for pancreatic islets culture; (B) Setup used for dynamic culture in biochip.


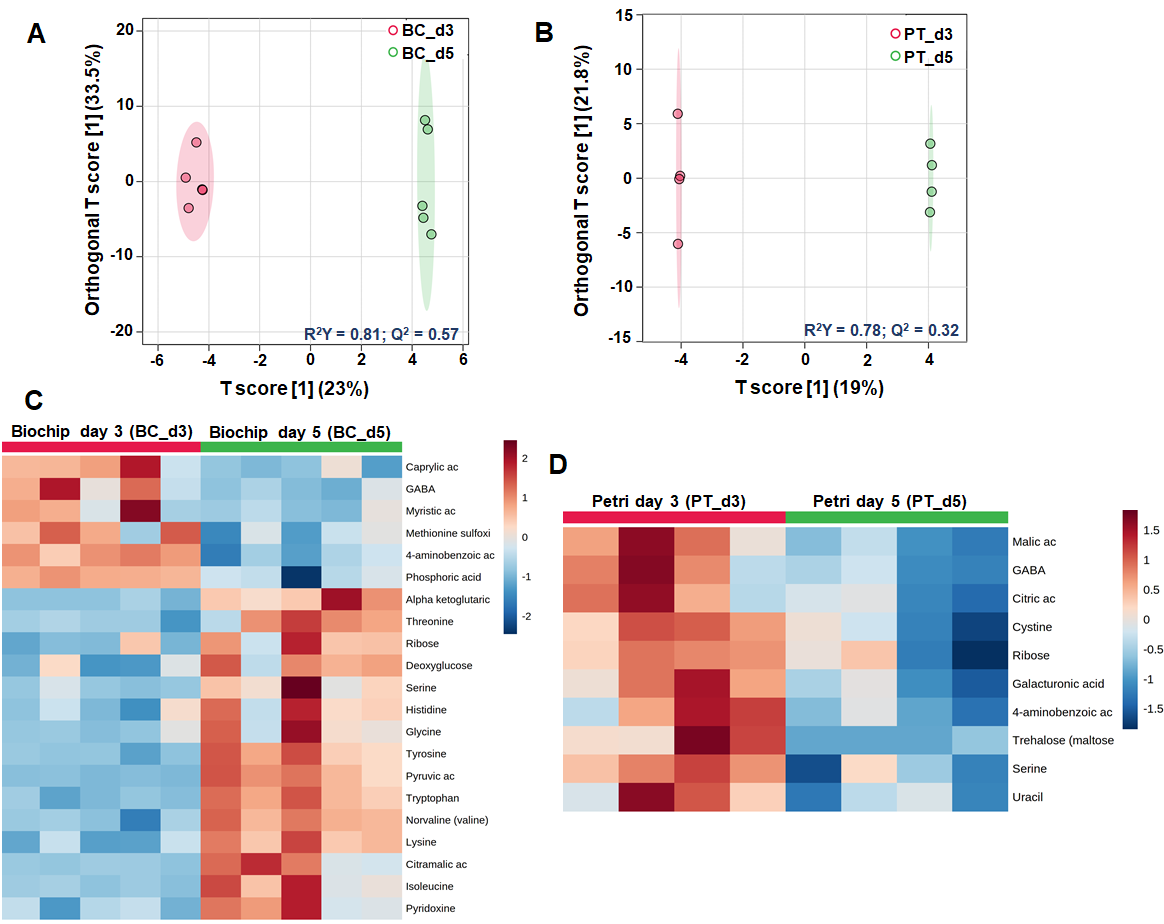


**Figure S2.** Comparison of days 3 and 5 in each culture modes. (A and B) OPLS-DA score plots of day 3 and day 5 comparisons in both modes of culture, (BC_d3 vs. BC_d5) and (PT_d3 vs. PT_d5), respectively; (C and D) heatmap of metabolites significantly modulated in BC_d3 vs. BC_d5 and PT_d3 vs. PT_d5 comparisons, respectively. OPLS-DA: orthogonal projections to latent structures discriminant analysis; BC_d3 and BC_d5: biochip cultures after 3 and 5 days; PT_d3 and PT_d5: Petri cultures after 3 and 5 days.


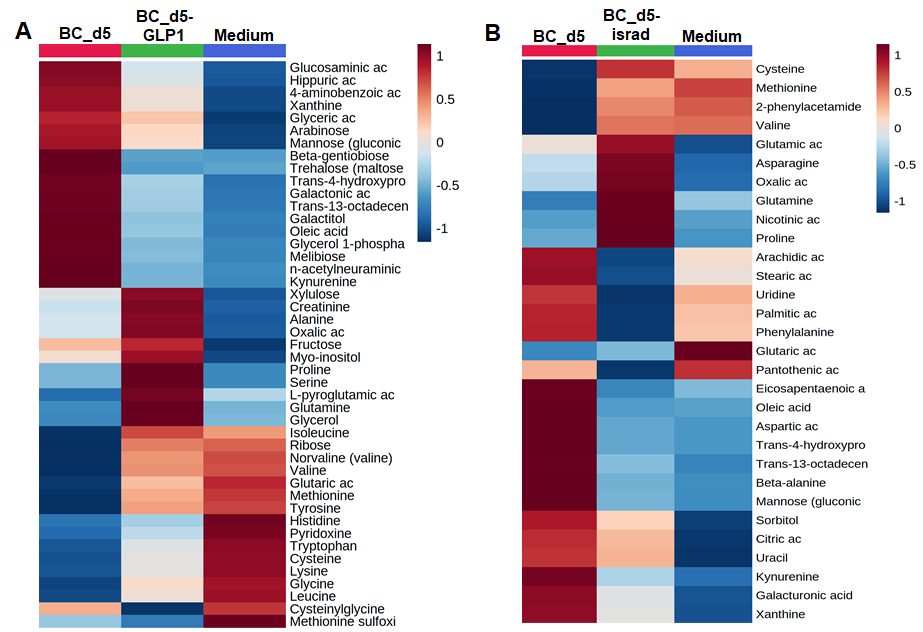


**Figure S3.** Heatmap of metabolites significantly modulated by GLP1 (A) and isradipine (B) treatments in biochip.


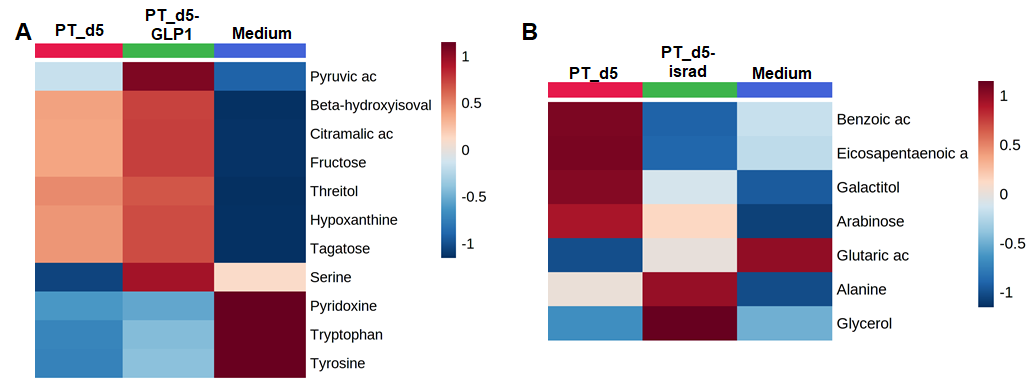


**Figure S4.** Heatmap of metabolites significantly modulated by GLP1 (A) and isradipine (B)treatments in Petri. PT_d5: Petri cultures after 5 days; PT_d5-GLP1: Petri culture treated with GLP1 after 5 days; PT_d5-israd: Petri culture treated with isradipine after 5 days; GLP1: glucagon-like peptide-1; israd: isradipine.


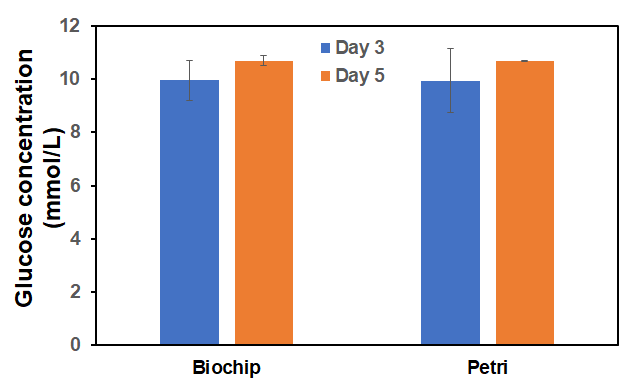


**Figure S5.** Glucose concentration in Petri and biochip at day 3 and 5 of culture.

**Table S1.** Set of metabolites identified in the samples by GC-MS.

| **Metabolite** | **PubChem ID** | **Metabolite** | **PubChem ID** |
| --- | --- | --- | --- |
| n-acetylneuraminic acid  Adipic acid  Alanine  Allantoin  Alpha ketoglutaric acid  4-aminobenzoic acid  Arachidic acid  Asparagine  Aspartic acid  Azelaic acid  Benzoic acid  Beta-alanine  Beta-gentiobiose  Beta-hydroxyisovalerate  Capric acid  Caprylic acid  Cholesterol  Citramalic acid  Citric acid  Citrulline  Creatinine  Cysteine  Cysteinylglycine  Cystine  Deoxyglucose  Eicosapentaenoic acid  Fructose  Galactitol  Galactonic acid  Galacturonic acid  gamma-aminobutyric acid GABA  Glucosaminic acid  Glucose  Glutamic acid  Glutamine  Glutaric acid  Glyceric acid  Glycerol  Glycerol 1-phosphate  Glycine  Glycolic acid  Heptadecanoic acid  Hexanoic acid  Hippuric acid  Histidine  Hypoxanthine  Isoleucine  Kynurenine  Lactic acid  Lauric acid | 445063  196  5950  204  51  978  10467  236  5960  2266  243  239  441422  69362  2969  379  304  439766  311  9750  588  594  65270  67678  439268  446284  5984  11850  128869  439215  119  73563  24749  33032  738  743  439194  753  754  750  757  10465  8892  464  6274  790  791  161166  107689  3893 | Leucine  Lysine  Arabinose  Malic acid  Mannose  Melibiose  Methionine  Methionine sulfoxide  1-methyl nicotinamide  Myo-inositol  Myristic acid  Nicotinic acid  Norvaline  Oleic acid  Ornithine  Oxalic acid  Palmitic acid  Pantothenic acid  2-phenylacetamide  Phenylalanine  3-phosphoglycerate  Phosphoric acid  Proline  Pyridoxine  L-pyroglutamic acid  Pyruvic acid  Ribose  Salicylic acid  Serine  Sorbitol  Stearic acid  Succinic acid  Threitol  Trehalose  Threonic acid  Threonine  Tagatose  Trans-4-hydroxyproline  Trans-13-octadecenoic acid  Triethanolamine  Tryptophan  Tyrosine  Uracil  Urea  Uric acid  Uridine  Valine  Xanthine  Xylitol  Xylulose | 6106  5962  66308  92824  18950  440658  6137  158980  457  892  11005  938  65098  445639  6262  971  985  6613  7680  994  439183  1004  145742  1054  7405  1060  993  338  5951  5780  5281  1110  169019  7427  5460407  6288  2724552  810  6161490  7618  6305  6057  1174  1176  1175  6029  6287  1188  6912  439204 |
